# Primary oesophageal melanoma - a case report

**DOI:** 10.1186/1477-7819-12-77

**Published:** 2014-03-31

**Authors:** Eric J Song, Richard A Scolyer, Diona L Damian, John F Thompson

**Affiliations:** 1 Discipline of Physiology, Faculty of Medicine, The University of Sydney and Bosch Institute, N543 Anderson Stuart Bldg (F13), Sydney, NSW 2006, Australia; 2 Department of Anatomical Pathology, Gosford Hospital, Holden St, Gosford, NSW 2250, Australia; 3 Tissue Pathology and Diagnostic Oncology, Royal Prince Alfred Hospital, Camperdown, NSW 2050, Australia; 4 Discipline of Pathology, Sydney Medical School, The University of Sydney, Sydney, NSW 2006, Australia; 5 Discipline of Dermatology, The University of Sydney at Royal Prince Alfred Hospital, Camperdown, NSW 2006, Australia; 6 Discipline of Surgery, Sydney Medical School, The University of Sydney, Sydney, NSW 2006, Australia; 7 Melanoma Institute Australia, North Sydney, NSW 2065, Australia; 8 Melanoma and Surgical Oncology, Royal Prince Alfred Hospital, Camperdown, NSW 2006, Australia

## Abstract

Primary upper gastrointestinal tract melanoma is a rare but well recognised entity, with a poor prognosis because of delay in diagnosis. Furthermore, it may be difficult to determine whether a gastrointestinal melanoma represents a metastasis or a primary tumour. We report a 67-year-old man with a primary oesophageal melanoma, treated with surgical resection, who remains disease-free two years post resection.

## Background

Most melanomas involving the gastrointestinal tract represent metastasis from known or unknown cutaneous or extracutaneous primary tumours. Primary upper gastrointestinal tract melanoma is rare and carries a poor prognosis [1]. The first suspected, but not histologically confirmed, case of primary oesophageal melanoma was reported in 1906 by Bauer [2]. Garfinkle reported histological evidence of a melanocytic naevus (as a presumed precursor lesion) adjacent to a primary gastroesophageal melanoma in 1956 [3], following Allen and Spitz’s suggestion of histological features required for such diagnosis [4]. Numerous case reports have been published since, but in many reports the documented clinical and pathological evidence indicating primary oesophageal melanoma was very limited, raising doubts about the true nature of some of these cases. The *c-KIT* mutation status for example, was reported in only four cases of primary oesophageal melanoma out of 337 cases reviewed in a recent report [1]. In this report, we document the clinical, pathological (including somatic mutation status), treatment and follow up details of a primary oesophageal melanoma.

## Case presentation

**Figure 1:**
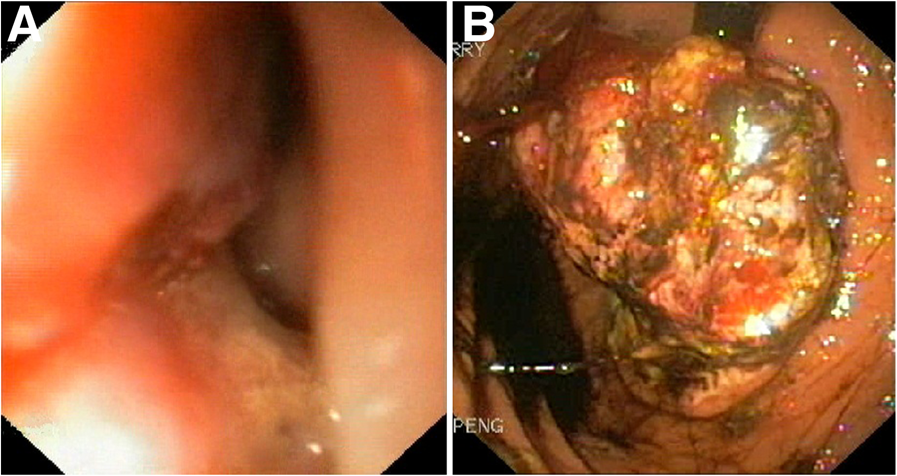
**Gastroscopic findings. (A)** Gastroscopy revealed an irregularly thickened, pigmented lesion at the distal end of the oesophagus. A polypoid tumour mass protruding through the gastroesophageal junction was seen **(B)** when viewed from the stomach.

A 67-year-old Singaporean man of Fitzpatrick’s skin type III [5], with Chinese ancestry, few naevi and only minimal previous sun exposure, presented to his family physician with a two-year history of persistent and progressively worsening left-sided chest pain, which began following a fall. His symptoms did not respond to analgesics, NSAID drugs or muscle relaxants, and investigations for cardiovascular causes of his pain were negative. In July 2009, a gastroscopy was performed which revealed a plaque with patchy, brownish discoloration arising from the distal oesophagus and extending into the gastric cardia as a polypoid mass (Figure 1).

**Figure 2:**
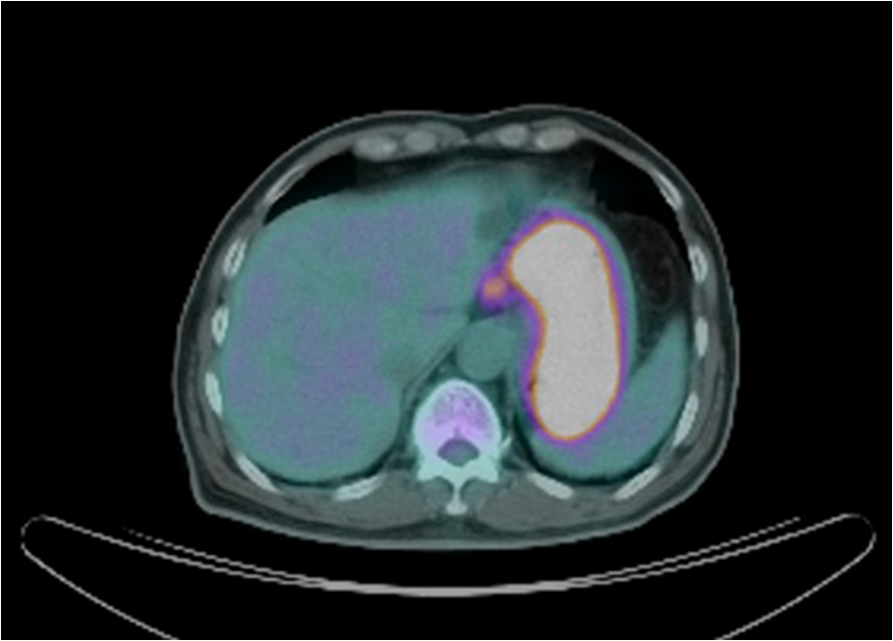
Positron emission tomography coupled with computerized tomography showed cardia and gastric wall thickening and a lymph node with strongly positive isotope uptake along the lesser curvature.

A tissue biopsy showed a poorly differentiated malignant tumour with morphological and immunohistochemical features consistent with melanoma (S-100 protein, Melan A/MART-1 and HMB45/Gp100 all positive). Full body positron emission tomography coupled with a non-contrast computerized tomography scan (PET-CT) showed extensive thickening of the oesophageal and gastric walls, with several enlarged perigastric lymph nodes strongly suggestive of malignancy (Figure 2). Thorough skin and eye examinations did not reveal any melanoma or suspicious lesion.

**Figure 3:**
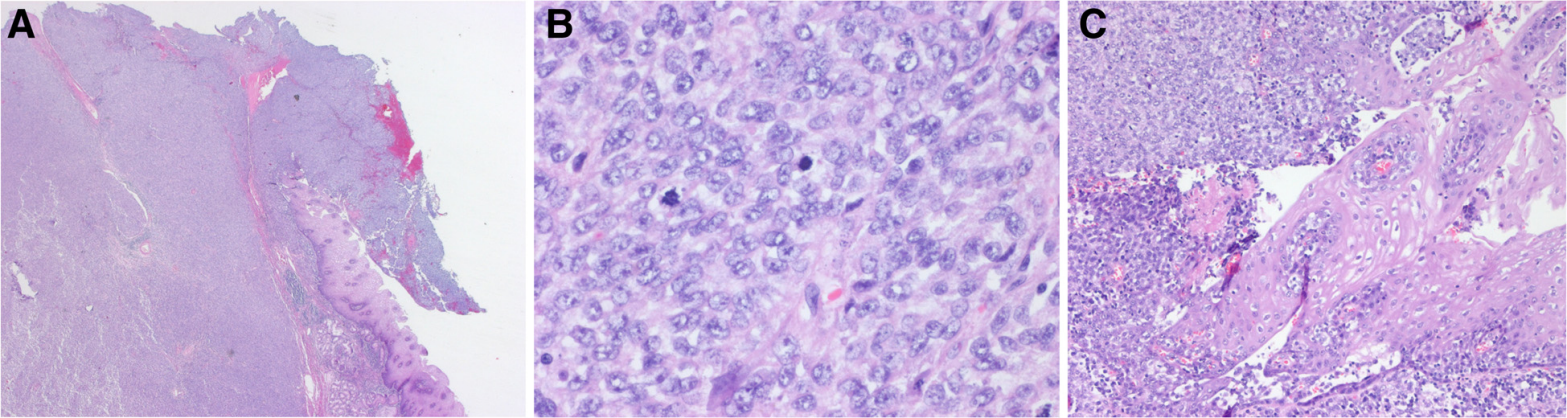
**Histopathologic findings.** Histopathology showed an exophytic and endophytic tumour involving the distal oesophagus **(A)**, comprised of large, pleomorphic tumour cells with frequent mitoses (15/mm^2^) **(B)**. Melanoma also involved the oesophageal squamous mucosa consistent with *in situ* disease **(C)**.

Total gastrectomy and partial oesophagectomy with lymph node clearance was performed. Conventional Roux-en-Y oesophagojejunostomy with distal jejunojejunostomy was used for reconstruction. Pathology showed a melanoma with a maximal thickness of 4 cm involving the distal oesophagus, extending into the gastric submucosa (Figure 3). It had a maximal diameter of 11 cm and extended through the muscularis propria to the serosa. The tumour was composed of confluent sheets of pleomorphic, rounded or spindle cells with enlarged nuclei, prominent nucleoli, and frequent mitoses (15/mm^2^) (Figure 3). Melanoma was focally present in the oesophageal squamous mucosa overlying the invasive tumour consistent with primary rather than secondary disease (Figure 3). Resection margins were clear.

Seven of ten nodes from the lesser curvature of the stomach were positive for melanoma, staining positively for Melan A, S-100 and HMB45 on immunohistochemistry, with no evidence of extranodal spread. Molecular testing revealed no somatic mutations in *BRAF* exon 15 or *c-KIT* exons 11, 13 or 17. On the basis of the absence of prior or concurrent melanoma elsewhere, the presence of melanoma in the squamous epithelium and the pattern of spread of the tumour to local lymph nodes, a diagnosis of primary melanoma of the oesophagus, with regional nodal metastasis (AJCC stage IIIc) was rendered. The patient was followed up with PET-CT post resection. There has been no clinical or radiological evidence of recurrent local or distant disease, and he remains well two years postoperatively.

## Discussion

A diagnosis of primary extracutaneous melanoma should not be rendered without first considering whether the tumour may represent a metastasis from an unrecognized or misdiagnosed melanoma [1]. Therefore a thorough history and examination is required to exclude a previous or synchronous cutaneous or extracutaneous primary melanoma elsewhere, including a regressed lesion or a previously removed melanocytic lesion misdiagnosed as a naevus. The presence of melanoma involving the mucosa overlying and adjacent to an invasive mucosal melanoma is also evidentiary support of a primary mucosal origin because it suggests *in situ* disease. However, this is not definitive because metastatic melanoma can show epithelial tropism and mimic a primary tumour histologically. Because it usually presents clinically at a locally advanced stage, the surface mucosa is usually ulcerated in primary oesophageal melanoma and hence an *in situ* melanoma component may not be identifiable. Finally, a credible diagnosis of primary oesophageal melanoma also requires that the pattern of any spread of the disease is consistent with the primary site of the tumour.

The occurrence of primary melanomas in mucosal sites has long been recognised. Melanocytes have been identified in normal individuals in most of these mucosal sites, including the oesophagus [6], providing a plausible histogenetic basis for the origin of these melanomas.

Risk factors specific for primary gastrointestinal melanoma are not clear. A recent review suggested that risk factors for cutaneous melanoma such as skin type, family history or sun exposure did not appear to be important for extracutaneous melanoma [1]. The clinical features of those patients reported in the literature to have oesophageal melanoma were similar to those of patients with oesophageal malignancies in general: most patients were men in their 60s or 70s [1]. Interestingly, primary oesophageal melanoma, like primary oesophageal carcinoma, occurs more frequently in Asian individuals compared to Caucasians [1,7]. However, other risk factors for oesophageal cancer such as gastroesophageal reflux disease, Barrett’s oesophagus, smoking, obesity and diet have not been implicated as risk factors for primary oesophageal melanoma. The estimated disease frequency was 0.1 to 0.2% of all oesophageal malignancies [1], with reported two- and five-year survival rates of 24 and 4.5%, respectively [1].

Activating mutations or gene amplification of the tyrosine kinase receptor *c-KIT* are present in 10 to 15% of acral and mucosal melanomas [8]. Langer *et al.* examined ten cases of primary oesophageal melanomas and found two cases with *c-KIT* mutations [9]. No *c-KIT* mutation was identified in our patient. Because mutations in *c-KIT* (and *BRAF*) are now being successfully exploited by new targeted therapies, it would appear appropriate to perform relevant mutation testing of primary extracutaneous melanoma in patients in whom systemic treatment is being considered.

The treatment of choice for primary oesophageal melanoma is radical surgical excision. Prognosis is generally very poor, with the principal determinant of outcome being the presence or absence of local lymph node metastases.

## Conclusion

Primary melanomas of the gastrointestinal tract are rare. Diagnosis requires exclusion of a metastasis through careful evaluation of the clinical history, physical examination, and pathological evaluation. Although the prognosis is poor, surgery for selected patients with resectable disease offers the best chance of cure. Recently developed targeted therapies may improve the therapeutic options for patients requiring systemic therapies for advanced stage disease.

### Consent

Written informed consent was obtained from the patient for publication of this Case report and any accompanying images. A copy of the written consent is available for review by the Editor-in-Chief of this journal.

## Abbreviations

NSAID: Non steroidal anti-inflammatory drug; PET: Positron emission tomography; CT: Computerized tomography; HMB: Human Melanoma Black; AJCC: American Joint Committee on Cancer.

## Competing interests

The authors declare that they have no competing interests.

## Authors’ contributions

ES performed literature review and prepared the initial manuscript draft including the background and case presentation. RS provided the slide photographs, the pathological evidence from which the pathological diagnosis was made and contributed to editing the manuscript. DD contributed to drafting and editing the manuscript, and JT contributed to editing the manuscript. All authors read and approved the final manuscript.
